# Enhancement of superconductivity under pressure and the magnetic phase diagram of tantalum disulfide single crystals

**DOI:** 10.1038/srep31824

**Published:** 2016-08-18

**Authors:** M. Abdel-Hafiez, X.-M. Zhao, A. A. Kordyuk, Y.-W. Fang, B. Pan, Z. He, C.-G. Duan, J. Zhao, X.-J. Chen

**Affiliations:** 1 Center for High Pressure Science and Technology Advanced Research, Shanghai, 201203, China; 2 Faculty of science, Physics department, Fayoum University, 63514-Fayoum- Egypt; 3 Department of Physics, South China University of Technology, Guangzhou 510640, China; 4 Institute of Metal Physics, National Academy of Sciences of Ukraine, 03142 Kyiv, Ukraine; 5 Key Laboratory of Polar Materials and Devices, Ministry of Education, East China Normal University, Shanghai 200241, China; 6 State Key Laboratory of Surface Physics and Department of Physics, Fudan University, Shanghai 200433, China; 7 Collaborative Innovation Center of Advanced Microstructures, Fudan University, Shanghai 200433, China

## Abstract

In low-dimensional electron systems, charge density waves (CDW) and superconductivity are two of the most fundamental collective quantum phenomena. For all known quasi-two-dimensional superconductors, the origin and exact boundary of the electronic orderings and superconductivity are still attractive problems. Through transport and thermodynamic measurements, we report on the field-temperature phase diagram in 2*H*-TaS_2_ single crystals. We show that the superconducting transition temperature (*T*_*c*_) increases by one order of magnitude from temperatures at 0.98 K up to 9.15 K at 8.7 GPa when the *T*_*c*_ becomes very sharp. Additionally, the effects of 8.7 GPa illustrate a suppression of the CDW ground state, with critically small Fermi surfaces. Below the *T*_*c*_ the lattice of magnetic flux lines melts from a solid-like state to a broad vortex liquid phase region. Our measurements indicate an unconventional *s*-wave-like picture with two energy gaps evidencing its multi-band nature.

For more than four decades, one of the major subjects in condensed matter physics has been the coexistence of the charge density wave (CDW) order and superconductivity in transition metal dichalcogenides (TMDs)^1^,^2^. In CDW materials such a coupling between the electrons and the soft-phonon mode describes the phase transition from the CDW to a normal state^3^. The superconducting transition temperature (*T*_*c*_) increases while the CDW lock-in temperature falls by doping^4^, critical thicknesses^5^, or by external pressure^6^,^7^,^8^. Recently, Klemm^9^ has shown that most of the pristine TMDs are highly unconventional in comparison with conventional superconductors. Amongst many TMD materials, 2*H*-TaS_2_ (*H*: hexagonal, see Methods, Extended Data Fig. 1) becomes superconducting at ambient pressure and without doping^4^. So far, this compound is one of the very few materials where a chiral and polar charge-ordered phase is suggested to exist^10^,^11^. Based on scanning tunneling microscopy measurements, the nodal gap structure of a single-layer material has recently been proposed^12^. Moreover, the lack of agreement on the electronic properties of 2*H*-TaS_2_, the information on its magnetic properties and, the Abrikosov vortex dynamics, is also missing up to now^13^. Therefore, the appearance of superconductivity in 2*H*-TaS_2_ in the presence of a CDW is of great interest. This has motivated us to study the low temperature-field dependencies of both transport and thermodynamics in the normal and superconducting states of 2*H*-TaS_2_ single crystals to determine their superconducting properties.

## Transport Measurements

**Figure 1:**
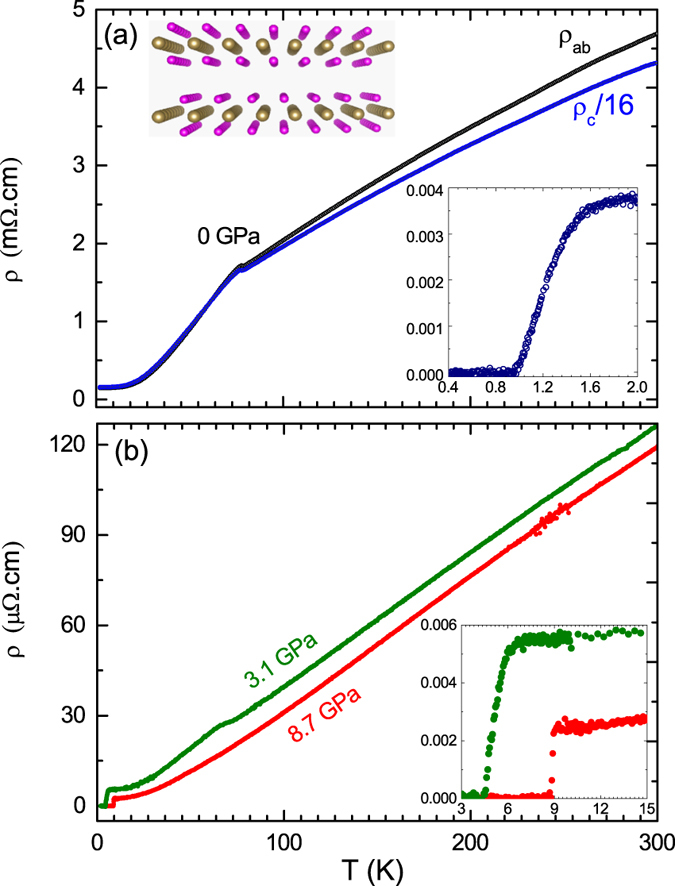
Transport measurements 2*H*-TaS_2_ at ambient and high pressures. (**a**) Temperature dependence of in-plane and out-of-plane resistivities at ambient pressure. The lower inset presents a zoom of the in-plane resistivity data around *T*_*c*_. The upper inset shows an expanded layered structure of 2*H*-TaS_2_. The 2*H* form is based on edge sharing TaS_6_ trigonal prisms. Each layer of TaS_2_ has a strongly bonded 2D S-Ta-S layers, with Ta in either trigonal prismatic or octahedral coordination with S. The chemical bonding within the S-Ta-S layers are covalently bound. (**b**) Temperature dependence of the in-plane electrical resistivity in zero-field at 3.1 GPa and 8.7 GPa. The inset represents a zoom of the in-plane resistivity data with a very sharp superconducting transition and the *T*_*c*_ enhances up to 9.15 K at 8.7 GPa.

**Figure 2:**
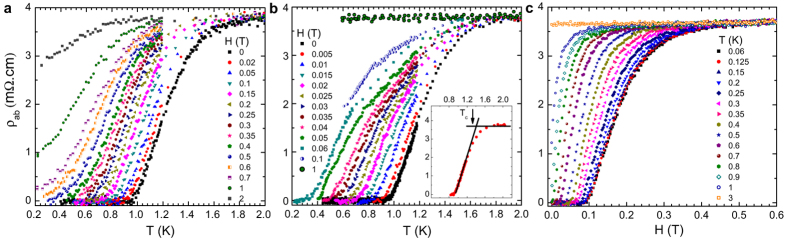
Low temperature and magnetic field dependencies of 2*H*-TaS_2_ resistivity. (**a**) The temperature dependence of *ρ*_*ab*_ at different magnetic fields for *H*||*ab* and *H*||*c*. (**b**) The inset shows the criterion for determining the *T*_*c*_ at 0.005 T. (**c**) The magnetic field dependence of the in-plane resistivity *ρ*_*ab*_ for *H*||*c*.

The temperature dependencies of the in-plane and out-of-plane zero-field resistivity (*ρ*_*ab*_ and *ρ*_*c*_) are shown in Fig. 1(a). Both *ρ*_*ab*_ and *ρ*_*c*_ exhibit a prominent CDW anomaly at 76 K (see The Methods, Extended Data Fig. 2). A parameter often used to characterize the interlayer coupling, is the anisotropy of the resistivity *ρ*_*c*_/*ρ*_*ab*_. The largest anisotropy ratio found here is *ρ*_*c*_/*ρ*_*ab*_ ~ 16 just above the *T*_*c*_. We noticed in particular that the anisotropy ratio is almost temperature independent. This anisotropy ratio behavior suggests that the in-plane and out-of-plane transport in 2*H*-TaS_2_ share the same scattering mechanism. Upon lowering the temperature below the CDW transition, the resistivity displays a drop to zero as shown in the inset of Fig. 1(a). The detailed magnetic field and temperature dependencies of *ρ*_*ab*_(*H*) at various temperatures ranging from, 60 mK to 3 K with the field direction parallel to the *c*-plane of the crystal, are presented in Fig. 2. At low temperatures, the curves are almost parallel to each other in the transition region. With increasing magnetic fields, the onset of superconductivity shifts to lower temperatures gradually. The suppression of superconductivity with a magnetic field applied along the *c*-direction is more obvious than that in the *H*||*ab* configuration, indicating a high anisotropy for a low *T*_*c*_ in 2*H*-TaS_2_.

It is worth mentioning that the *T*_*c*_ in resistivity at ambient pressure is anomalously wide. It is about 0.62 K from the onset *T*_*c*_ value to the zero resistivity value of the *T*_*c*_ at 0.98 K, i.e. about 50% of the *T*_*c*_. This anomalous 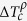 could have several sources: chemical or electronic inhomogeneity, fluctuations, or vortex effects. Inhomogeneity is indeed expected to widen the transition of this compound because studies show that even a small concentration of dopants enhance the *T*_*c*_ dramatically^4^,^14^. This is why superconductivity above 1 K in nominally pure 2*H*-TaS_2_ is explained by a small Ta excess or by the presence of sub 1% quantities of impurity atoms^4^. An intrinsic electronic inhomogeneity related to inhomogeneous CDW is quite possible as the chiral CDW reported for this compound supposes a domain structure^4^ which is inline with the observed narrowing of the *T*_*c*_ with CDW destruction, for example with doping by Ni^14^. On the other hand, both kinds of inhomogeneity could also affect the width of the transition in heat capacity; however that is only about 0.2 K, much narrower than the 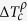. This suggests that the effects of these inhomogeneities are limited by 0.2 K, while the rest of the 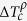 is related to fluctuations and/or vortices. The dissipative vortex motion could either be due to the flux flow through low pinning centers or due to the free motion of individual vortices in the vortex liquid state. Since in our resistivity experiments we used the lowest current 0.1*μ*A and the 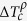 was not sensitive to its small enhancement, we may consider the vortex liquid state as the most probable mechanism of widening the transition, similarly to Cu_*x*_TiSe_2_^15^. The vortex liquid can be considered a result of fluctuations in the vortex lattice below the *T*_*c*_, while fluctuations in the superconducting order parameter lead to the appearance of preformed pairs above the *T*_*c*_. The measurement of both fluctuation regions is the Ginzburg number *Gi* = *δT*/*T*_*c*_, which is usually extremely small for the low-temperature superconductors *Gi* ~ (*T*_*c*_/*E*_*F*_)^4^ ~ 10^−12^–10^−14^, even for two-dimensional ones, for which *Gi* ~ *T*_*c*_/*E*_*F*_ or *τ*^−1^/*E*_*F*_ for the clean and dirty limits respectively^16^. Here *δT* is the range of temperatures in which fluctuation corrections are relevant, and *τ*^−1^ is the quasiparticle scattering rate at the Fermi energy (*E*_*F*_). However, in the CDW state, the reconstructed FS may have small and very shallow pockets for which the *E*_*F*_ could not be much larger than *T*_*c*_ or 1/*τ*^17^. Therefore, the broadening of the *T*_*c*_ due to the interplay with CDW is further supported by the sharp *T*_*c*_ after the suppression of the CDW upon compression.

## Enhancement of Superconductivity Upon Compression

In low-dimensional electron systems, CDW and superconductivity are two of the most fundamental collective quantum phenomena^1^,^2^. Unconventional superconductivity is nearly always found in the vicinity of another ordered state, such as antiferromagnetism, CDW, or stripe order. This suggests a fundamental connection between superconductivity and fluctuations in some other order parameter^18^. To better understand this connection, we used high-pressure resistivity to directly study the CDW order in 2*H*-TaS_2_. The effect of pressure on 2*H*-TaS_2_ is presented in Fig. 1(b). Upon 3.1 GPa, the CDW slightly shifts to 69 K. The effects of 8.7 GPa illustrate a suppression of the *T*_*CDW*_. In addition, a very sharp drop in resistivity indicates the onset of superconductivity and dramatically enhances the modest *T*_*c*_ to ~9.15 K upon 8.7 GPa. Similarly to recently reported data^19^, our resistance measurements show that the *T*_*c*_ increases from temperatures below 1 K up to 8.5 K at 9.5 GPa. Additionally, the authors observed a kink in the pressure dependence of *T*_*CDW*_ at about 4 GPa that they attributed to the lock-in transition from an incommensurate CDW to a commensurate CDW. Above this pressure, the commensurate *T*_*CDW*_ slowly decreases, coexisting with superconductivity within our full pressure range. These observations show that the enhancement in superconductivity is due to the consequent changes of Fermi surface (FS) upon compression. However, this is not direct evidence that confirms where such features act on superconductivity independently of the CDW. In the CDW state, a gap opens up over part of the FS in the direction of the *q* vectors of the CDW^8^. This reduces the average density of states at the FS. Upon compression, *T*_*CDW*_ is suppressed. The amplitude of the CDW lattice distortion also suppresses, thus gradually restoring the FS and increasing the *T*_*c*_. Therefore, one can see that both superconductivity and the CDW involve widely different parts of the FS associated with the absence of or small interband correlations. It is worth noting that superconductivity in 2*H*-NbSe_2_ is only moderately affected by pressure^20^,^21^ and the CDW already disappears at 5 GPa^20^,^22^. The weak pressure dependence of the *T*_*CDW*_ at higher pressures indicates that the CDW in this pressure range is remarkably robust to a reduction in the lattice parameters^19^. Very recently^23^, in 2*H*-NbSe_2_ the rapid destruction of the CDW under pressure was found to be related to the quantum fluctuations of the lattice renormalized by the anharmonic part of the lattice potential. In addition, the connection between CDWs and superconductivity arises from the fact that high-energy optical phonon modes have a strong contribution to the Eliashberg function, whereas the low-energy longitudinal acoustic mode that drives the CDW transition barely contributes to superconductivity

## Specific Heat Measurements

**Figure 3:**
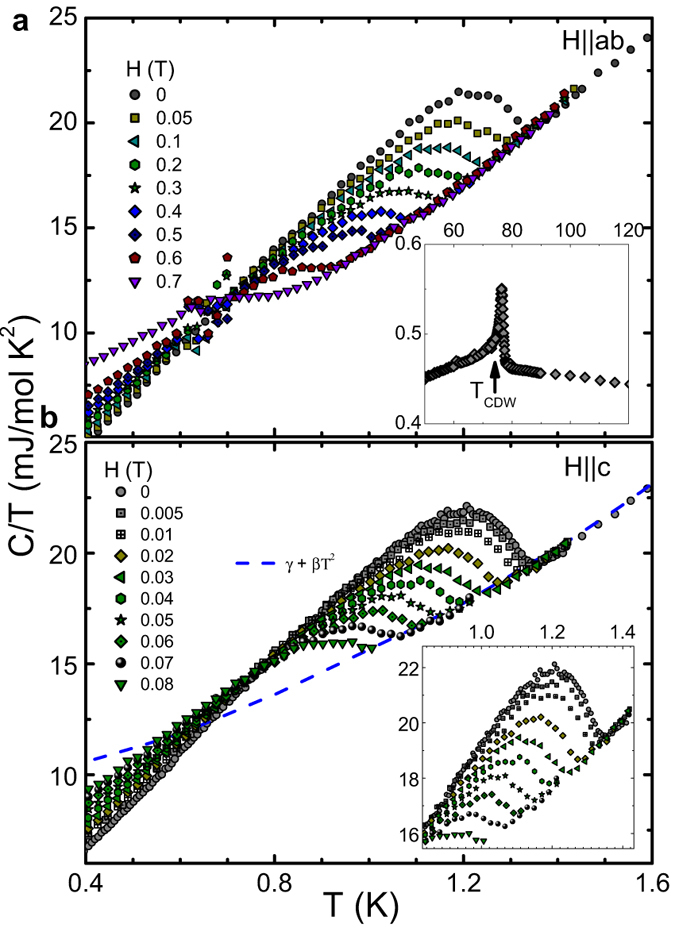
Temperature dependence of 2*H*-TaS_2_ specific heat. *T*-dependence of the specific heat in various applied magnetic fields parallel to the *ab* axis (**a**) and parallel to the *c* plane (**b**). The dashed line in (**b**) is the fitting below 2.5 K by using *C*_p_ = *γ*_*n*_*T* + *βT*^3^. The inset in (**b**) shows a close-up of the superconducting state while the inset of (**a**) presents the CDW state.

To further elucidate the bulk superconductivity in 2*H*-TaSe_2_, we performed heat capacity studies down to 70 mK. Figure 3 summarizes the *T*-dependence of the specific heat data in various magnetic fields applied parallel and perpendicular to the ab plane. We observed a clear sharp anomaly at *T*_*c*_ = 1.4 K, close to that determined by our resistivity measurements. The specific heat jump systematically shifted to lower temperatures upon the application of magnetic fields. Our data of small fields close to the *T*_*c*_ shows the evolution of a small fluctuation, peak, overlapped with the specific-heat jump [see inset of Fig. 3(b)]. On the other hand, both kinds of chemical or electronic inhomogeneity should also affect the width of the transition in heat capacity, however that is only about 0.2 K, much narrower than the 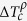. This suggests that the effects of inhomogeneities are limited by 0.2 K, while the rest of 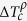 could be related to fluctuations and/or vortices. A clear maximum of specific heat data at 76 K, typically found in 2*H*-TaS_2_ which is weakly first-order, is an indication of the CDW transition [see the inset of Fig. 3(a)]. Note that there is no upturn (Schottky nuclear contribution) in the specific heat data measured to temperatures as low as 70 mK, thus, the zero-field specific heat above *T*_*c*_ can be well fitted to *C*_*p*_/*T* = *γ*_*n*_ + *βT*^2^, where *γ*_*n*_ and *β* are the electronic and lattice coefficients, respectively [see the dashed line in Fig. 3(b)]. The *γ*_*n*_ value is found to be around 8.8 mJ/mol K^2^, indicating that 2*H*-TaS_2_ in the CDW state is characterized by a modest density of states. This value agrees with the *γ*_*n*_ value found by refs 4 and 24 in which *C*_*p*_ was just measured between 1.8 and 10 K. The phononic coefficient *β* is found to be 0.35 mJ/mol K^4^. Using the relation *θ*_*D*_ = (12*π*^4^*RN*/5*β*)^1/3^, we obtained the Debye temperature *θ*_*D*_ = 249(2) K, which is comparable with values reported by DiSalvo *et al*.^2^. From the determined *γn* value, we found that Δ*C*_el_/*γ*_n_*T*_c_ = 0.72. This value is smaller than the prediction of the weak coupling BCS theory (Δ*C*_el_/*γ*_n_*T*_c_ = 1.43) and comparable to that in the intercalated compound^24^. This indicates that the specific-heat data cannot be described by a simple BCS gap (see Methods, Extended Data Fig. 3). However, in a clean situation with negligible pair-breaking effects, the reduced jump in the specific heat compared to that of a single-band *s*-wave superconductor might be related to unconventional superconductivity with nodes and/or a pronounced multiband character with rather different partial densities of states and gap values^25^. In addition, evidence of coupling effects arises from the normalized discontinuity value of the specific-heat slopes at the *T*_*c*_, (*T*_*c*_/Δ*C*)(*dC*/*dT*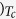. In the single-band weak coupling BCS theory this ratio is 2.64, whereas a value of 3.35 can be deduced in the two-band superconductor MgB_2_^26^. From our data, we obtained a value of (*T*_*c*_/Δ*C*)(*dC*/*dT*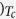 ~ 3.54, which is very close to MgB_2_.

## H - T Phase Diagram

**Figure 4:**
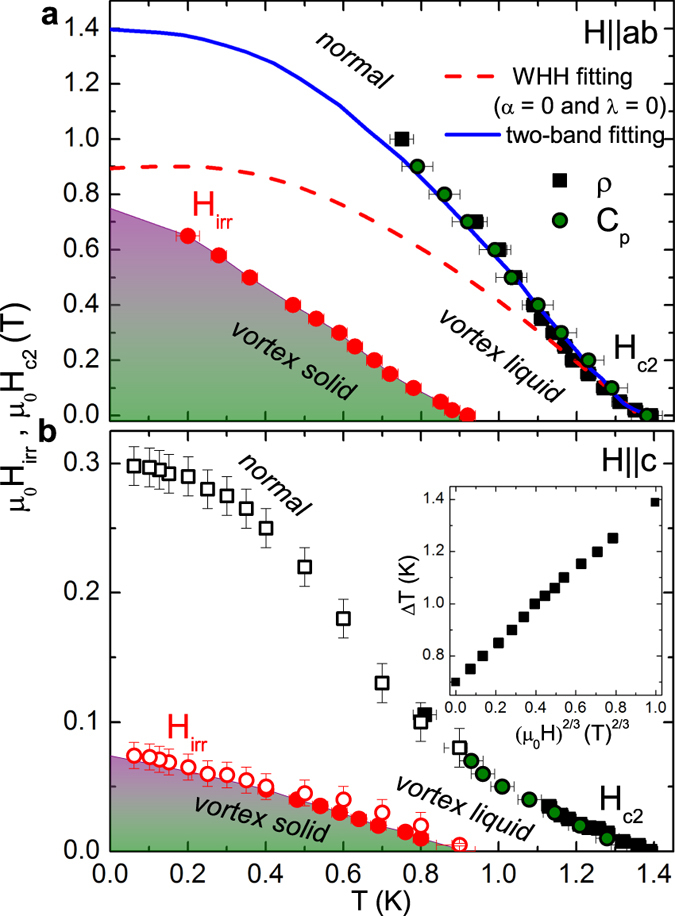
H - T phase diagram of 2*H*-TaS_2_. (**a**) The upper critical field *μ*_0_*H*_*c*2_ and the irreversible field, *μ*_0_*H*_*irr*_ for *H*||*ab* (**a**) and *H*||*c* (**b**). Open symbols in (**b**) are taken from *ρ*_*ab*_(*H*). The inset illustrates the transition width (Δ*T* vs. *μ*_0_*H*^2/3^). The dashed line is the linear fit.

The *H*_*c*2_ provides a valuable insight into the nature of the interaction responsible for the formation of Cooper pairs^25^,^27^,^28^. The temperature dependencies of *H*_*c*2_ and *H*_*irr*_ obtained from *C*(*T*, *H*) and *ρ*(*T*, *H*) with both *H*||*c* and *H*||*ab* are plotted in Fig. 4 for both orientations. Specific heat *T*_*c*_(*H*) values were deduced from the classical entropy conservation construction. The 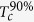 criteria of the normal state in resistivity was used to extract the *T*_*c*_ at each magnetic field. The irreversible magnetic field *H*_*irr*_ was obtained from the zero value of *T*_*c*_ in *ρ*_*ab*_ curves. However, the width of the resistive transition is shown in the inset of Fig. 4(b) and is proportional to *μ*_0_*H*^2/3^. This is inline with Tinkham’s theoretical prediction^29^ of the Δ*T* ∝ *μ*_0_*H*^2/3^. The large area between the *H*_*c*2_ and *H*_*irr*_ curves suggests that the vortex dissipation level is still low in this region. Moreover, the possible existence of a distinct *H*_*irr*_(*T*) far below *H*_*c*2_ is due to the fact that the vortex lattices are soft and easily melted into vortex liquid by the magnetic field or thermal fluctuations^30^. The zero-temperature values for 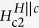 and 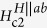 are estimated to be approximately 0.31 and 1.38 T, respectively. From those we estimated the anisotropic coherence length 
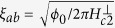
 = 32.6 nm, and *ξ*_*c*_ = 7.3 nm. One can also estimate the coherence length from the uncertainty principle and BCS model. From the Faber-Pippard ratio, *ξ* = 0.18*ħv*_*F*_/*k*_*B*_*T*_*c*_ = 260 nm, for *T*_*c*_ = 1.4 K and an average Fermi velocity *v*_*F*_ ≈ 1.5 eVÅ^17^, which is considered to be similar for 2*H*-TaSe_2_, 2*H*-NbSe_2_, and 2*H*-TaS_2_. This shows that both anisotropy and CDW effects on electronic structure should be taken into account. Furthermore, it has been reported^31^ that the field-induced antiferromagnetism can extend outside the effective vortex core region where the superconducting order parameter is finite. Such an extended magnetic order is expected to suppress the superconducting order parameter around vortices. This effect will enlarge the vortex core size, which in turn will suppress the *H*_*c*2_. The effective core size has been found to be around three times that of the coherence length in 2*H*-NbSe_2_^32^. From the behavior of *H*_c2_ vs. *T* for the different field orientations, we have calculated the anisotropy as 
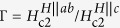
 = *ξ*_*ab*_/*ξ*_*c*_. The anisotropy Γ increases upon approaching the *T*_c_ and reaches about 4(1). This indicates that the orbital pair breaking also accounts for the suppression of superconductivity close to *T*_*c*_ in 2*H*-TaS_2_.

In the case of multi-band superconductivity^33^,^34^,^35^,^36^ the low-temperature *H*_*c*2_-curve may exceed the single-band Werthamer-Helfand-Hohenberg predictions^37^. However, a noticeable upward curvature in the *H*_*c*2_(*T*) observed in some compounds has been attributed to multiband effects^38^. Using typical renormalized Fermi velocities derived from preliminary ARPES-data^17^ and *T*_*c*_ = 1.4 K, one also estimates, that in principle by a two-band approach adopting *s*-symmetry^38^, the slope-value is: *H*_c2,*c*_ = 
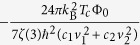
, where *c*_1_ → *c*_2_ → 1/2 and 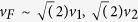 in the case of a dominant interband pairing results in -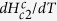 = 0.14 T/K near the *T*_*c*_ which is already very close to our experimentally determined value. By fitting it using the two-band theory^33^,^39^,^40^, one can obtain the band diffusivities *D*_1_, *D*_2_ and the intraband and interband coupling constants *λ*_11_, *λ*_12_, and *λ*_21_. The exact relations can be found in ref. 38. Using the band diffusivity ratio *η* = *D*_2_/*D*_1_ = 800, *λ*_11_ = 0.5, and *λ*_12_ = *λ*_21_ = 0.25, we fitted our data for 2*H*-TaS_2_. The obtained two-band fitting agrees well with the experimental data. To add more insight to the pairing symmetry for the 2*H*-TaS_2_ superconductor, we investigated the temperature dependence of the specific heat. The detailed electronic specific heat data and analysis are given in the Extended Data Fig. 3.

Summarizing, we have reported the first superconducting fluctuations investigation across the effect of pressure on the CDW state in 2H-TaS_2_. From an extensive thermodynamic study, we found a considerable broadening of the *T*_*c*_ at ambient pressure and its sharp transition at high pressures together with an unexpectedly broad region of vortex liquid phase in the vortex phase diagram. These results suggest the presence of the the superconducting fluctuations in the CDW state. Besides of a clear fundamental interest in our system, this finding can be used to control the fluctuations in quantum devices.

## Methods Summary

Low-temperature transport (down to 60 mK) and specific heat (down to 70 mK) measurements were performed using a dilution refrigerator. The conductance anisotropy in layered material single crystals is large therefore using traditional four-terminal methods to determine the resistivity along the *c* axis, *ρ*_*c*_, and in the *ab* plane, *ρ*_*ab*_, may be unreliable^41^. We used six terminals to determine each principal component of resistivity. In the latter method, the current was injected through the outermost contacts on one surface. Voltages were measured across the innermost contacts of each surface. The Laplace equation was then solved and inverted to find *ρ*_*c*_ and *ρ*_*ab*_^42^. In addition, this method allowed testing the sample homogeneity by permuting the electrodes which were used for the current and voltage^41^,^42^. Four contacts were used to measure the high-pressure in-plane resistivity. The investigated 2*H*-TaS_2_ single crystals were synthesized at hq graphene and were of high purity (>99.995%). The resistivity and specific heat measurements down to 0.4 K were measured in a Physical Property Measurement System (Quantum Design) with an adiabatic thermal relaxation technique.

### Online Content

Any additional Methods, Extended Data display items and Source Data are available in the online version of the paper; references unique to these sections appear only in the online paper.

## Additional Information

**How to cite this article**: Abdel-Hafiez, M. *et al*. Enhancement of superconductivity under pressure and the magnetic phase diagram of tantalum disulfide single crystals. *Sci. Rep.* **6**, 31824; doi: 10.1038/srep31824 (2016).

## Supplementary Material

Supplementary Information

## References

[b1] Wilson J. A. & Yoffe A. D. The transition metal dichalcogenides discussion and interpretation of the observed optical, electrical and structural properties. Adv. Phys. 18, 193–335 (1969).

[b2] DiSalvo F. J., Schwall R., Geballe T. H., Gamble F. R. & Osiecki J. H. Superconductivity in layered compounds with variable interlayer spacings. Phys. Rev. Lett. 27, 310 (1971).

[b3] Littlewood P. B. & Varma C. M. Gauge-invariant theory of the dynamical interaction of charge density waves and superconductivity. Phys. Rev. Lett. 47, 811 (1981).

[b4] Wagner K. E. . Tuning the charge density wave and superconductivity in Cu_*x*_TaS_2_. Phys. Rev. B 78, 104520 (2008).

[b5] Yu Y. . Gate-tunable phase transitions in thin flakes of 1*T*-TaS_2_. Nat. Nano. 10, 270–276 (2015). 10.1038/nnano.2014.323 25622230

[b6] Chi Z.-H. . Pressure-induced metallization of molybdenum disulfide. Phys. Rev. Lett. 113, 036802 (2014). 25083660 10.1103/PhysRevLett.113.036802

[b7] Sipos B. . From Mott state to superconductivity in 1*T*-TaS_2_. Nat. Mat. 7, 960–965 (2008). 10.1038/nmat2318 18997775

[b8] Berthier C., Molinie P. & Jerome D. Evidence for a connection between charge density waves and the pressure enhancement of superconductivity in 2*H*-NbSe_2_. Solid State Commun. 18, 1393–1395 (1976).

[b9] Klemm R. A. Pristine and intercalated transition metal dichalcogenide superconductors. Physica C. 514, 86–94 (2015).

[b10] Guillamon I. . Chiral charge order in the superconductor 2*H*-TaS_2_. New J. Phys. 13, 103020 (2011).

[b11] Wezel J. van. Polar charge and orbital order in 2*H*-TaS_2_. Phys. Rev. B 85, 035131 (2012).

[b12] Galvis J. A. . Zero-bias conductance peak in detached flakes of superconducting 2*H*-TaS_2_ probed by scanning tunneling spectroscopy. Phys. Rev. B 89, 224512 (2014).

[b13] Kordyuk A. A. Pseudogap from ARPES experiment: three gaps in cuprates and topological superconductivity. Low Temp. Phys./Fiz. Nizk. Temp. 41, 417 (2015).

[b14] Li L. . Superconductivity of Ni-doping 2*H*-TaS_2_. Physica C 470, 313–317 (2010).

[b15] Husaníková P. . Magnetization properties and vortex phase diagram of Cu_*x*_TiSe_2_ single crystals. Phys. Rev. B 88, 174501 (2013).

[b16] Larkin A. & Varlamov A. Theory of Fluctuations in Superconductors (Oxford University Press, Oxford, 2005).

[b17] Borisenko S. V. . Pseudogap and charge density waves in two dimensions. Phys Rev. Lett. 100, 196402 (2008). 18518466 10.1103/PhysRevLett.100.196402

[b18] Joe Y. I. . Emergence of charge density wave domain walls above the superconducting dome in 1*T*-TiSe_2_. Nat. Phy. 10, 421–425 (2014).

[b19] Freitas D. C. . Strong enhancement of superconductivity at high pressures within the charge-density-wave states of 2H-TaS_2_ and 2H-TaSe_2_. Phys Rev. B 93, 184512 (2016).

[b20] Coronado E. . Coexistence of superconductivity and magnetism by chemical design. Nat. Chem. 2, 1031 (2010). 21107366 10.1038/nchem.898

[b21] Suderow H. . Pressure Induced Effects on the Fermi Surface of Superconducting 2*H*-NbSe_2_. Phys. Rev. Lett. 95, 117006 (2005). 16197038 10.1103/PhysRevLett.95.117006

[b22] Feng Y. . Order parameter fluctuations at a buried quantum critical point. Proceedings of the National Academy of Sciences 109, 7224 (2012). 10.1073/pnas.1202434109 PMC3358829 22529348

[b23] Leroux M. . Strong anharmonicity induces quantum melting of charge density wave in 2H-NbSe2 under pressure. Phys. Rev. B 92, 140303(R) (2015).

[b24] Schlicht A. . Superconducting Transition Temperature of 2*H*-TaS_2_ Intercalation Compounds Determined by the Phonon Spectrum. J. Phys. Chem. B 105, 4867–4871 (2001).

[b25] Abdel-Hafiez M. . Specific heat and upper critical fields in Kfe_2_As_2_ single crystals. Phys. Rev. B 85, 134533 (2012).

[b26] Bouquet F., Fisher R. A., Phillips N. E., Hinks D. G. & Jorgensen J. D. Specific heat of Mg_11_B_2_: evidence for a second energy gap. Phys. Rev. Lett. 87, 047001 (2001). 11461636 10.1103/PhysRevLett.87.047001

[b27] Hunte F. . Two-band superconductivity in LaFeAsO_0.8_9F_0.11_ at very high magnetic fields. Nature 453, 903–905 (2008). 18509332 10.1038/nature07058

[b28] Abdel-Hafiez M. . Superconducting properties of sulfur-doped iron selenide. Phys. Rev. B 91, 165109 (2015).

[b29] Tinkham M. Resistive transition of high-temperature superconductors. Rev. Phys. Lett. 61, 1659 (1988). 10.1103/PhysRevLett.61.1658 10038862

[b30] Blatter G., Feigel’man M. V., Geshkenbein V. B., Larkin A. I. & Vinokur V. M. Vortices in high-temperature superconductors. Rev. Mod. Phys. 66, 1125 (1994).

[b31] Zhang Y., Demler E. & Sachdev S. Competing orders in a magnetic field: Spin and charge order in the cuprate superconductors. Phys. Rev. B 66, 094501 (2002).

[b32] Hartmann U., Golubov A. A., Drechsler T., Kupriyanov M., Yu. & Heiden C. Measurement of the vortex-core radius by scanning tunneling microscopy. Physica B 194, 387–388 (1994).

[b33] Gurevich A. Enhancement of the upper critical field by nonmagnetic impurities in dirty two-gap superconductors. Phys. Rev. B 67, 184515 (2003).

[b34] Golubov A. A. & Koshelev A. E. Upper critical field in dirty two-band superconductors: Breakdown of the anisotropic Ginzburg-Landau theory. Phys. Rev. B 68, 104503 (2003).

[b35] Koshelev A. E. & Golubov A. A. Mixed State of a Dirty Two-Band Superconductor: Application to MgB_2_. Phys. Rev. Lett. 90, 177002 (2003). 12786096 10.1103/PhysRevLett.90.177002

[b36] Koshelev A. E. & Golubov A. A. Why Magnesium Diboride Is Not Described by Anisotropic Ginzburg-Landau Theory. Rev. Phys. Lett. 92, 107008 (2004). 10.1103/PhysRevLett.92.107008 15089235

[b37] Werthamer N. R., Helfand E. & Hohenberg P. C. Temperature and purity dependence of the superconducting critical field, *H*_*c*2_. III. Electron spin and spin-orbit effects. Phys. Rev. 147, 295–302 (1966).

[b38] Gurevich A. Iron-based superconductors at high magnetic fields. Rep. Prog. Phys. 74, 124501 (2011).

[b39] Golubov A. A. . Specific heat of MgB_2_ in a one- and a two-band model from first-principles calculations. J. Phys.: Condens. Matter. 14, 1353–1360 (2002).

[b40] Dolgov O. V., Kremer R. K., Kortus J., Golubov A. A. & Shulga A. V. Thermodynamics of two-band superconductors: The case of MgB_2_. Phys. Rev. B 72, 024504 (2005).

[b41] Li Q. A. . Reentrant orbital order and the true ground state of LaSr_2_Mn_2_O_7_. Phys. Rev. Lett. 98, 167201 (2007). 17501455 10.1103/PhysRevLett.98.167201

[b42] Busch R., Ries G., Werthner H., Kreiselmeyer G. & Saemann-Ischenko G. New aspects of the mixed state from six-terminal measurements on Bi_2_Sr_2_CaCu_2_O_*x*_ single crystals. Phys. Rev. Lett. 69, 522 (1992). 10046960 10.1103/PhysRevLett.69.522

