# Supplementary material for: Enhancement of superconductivity under pressure and the magnetic phase diagram of tantalum disulfide single crystals
Source: Sci Rep. 2016 Aug 18;6:31824. doi: 10.1038/srep31824 (PMC4989151; doi:10.1038/srep31824)
Supplement: Supplementary Information [file srep31824-s1.pdf]

Supplementary Material for
Enhancement of superconductivity under pressure and the magnetic phase diagram of tantalum disulfide single crystals

M. Abdel-Hafiez,[1,2] X.-M. Zhao,[1,3] A. A. Kordyuk,[4] Y.-W. Fang,[5]
B. Pan,[6] Z. He,[1,6] C.-G. Duan,[5] J. Zhao,[6,7] and X.-J.Chen[1]

[1]*Center for High Pressure Science and Technology Advanced Research, Shanghai, 201203, China*
[2]*Faculty of science, Physics department, Fayoum University, 63514-Fayoum- Egypt*
[3]*Department of Physics, South China University of Technology, Guangzhou 510640, China*
[4]*Institute of Metal Physics, National Academy of Sciences of Ukraine, 03142 Kyiv, Ukraine*
[5]*Key Laboratory of Polar Materials and Devices, East China Normal University, Shanghai 200241, China*
[6]*State Key Laboratory of Surface Physics and Department of Physics, Fudan University, Shanghai 200433, China*
[7]*Collaborative Innovation Center of Advanced Microstructures, Nanjing 210093, China*

SAMPLE CHARACHTERIZATION

Natural or synthetic TaS_2 crystallize in two different polytypisms, $2H$ (space group: P63/mmc) and $3R$ (space group: $R3m$), with the former being the dominating one. In both phases the Ta atom in one layer is aligned over

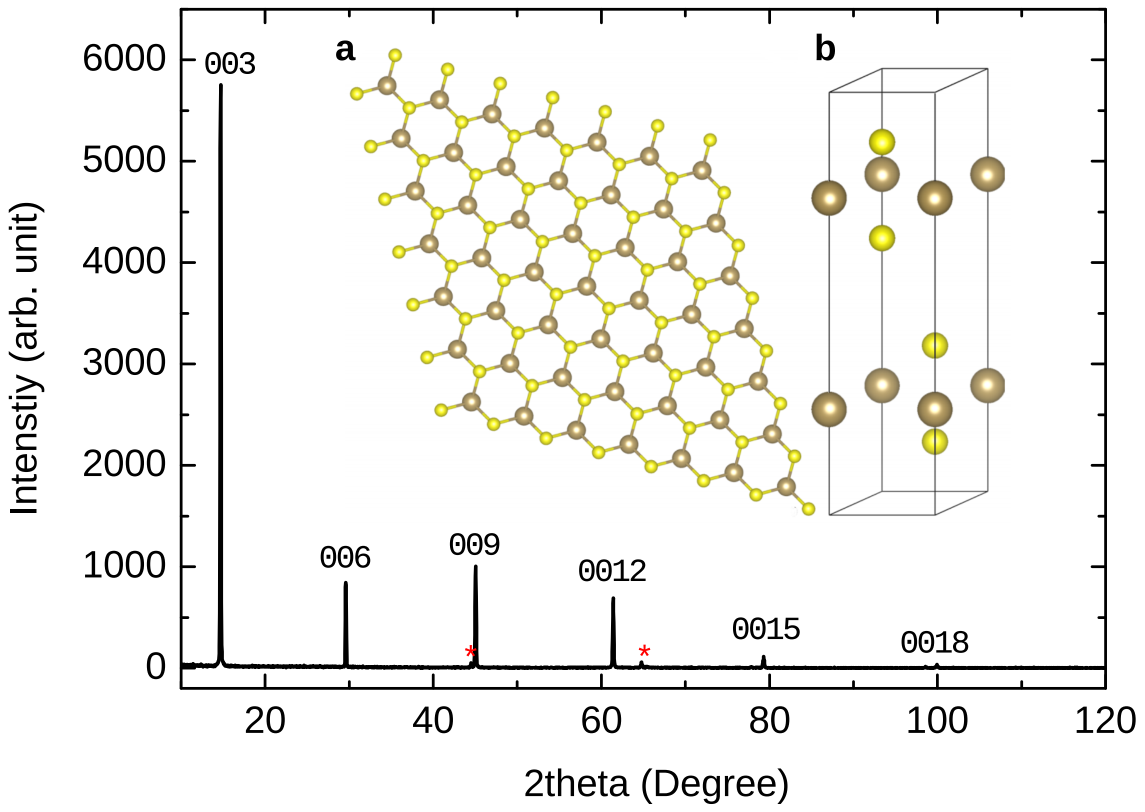

Extended Data Figure 1: Crystal Structure and X-ray single crystal diffraction of 2H-TaS$_2$: X-ray diffraction patterns along c-axis of 2H-TaS$_2$. Note that a red asterisk is the background signal from the vacuum chuck. The insets **a** presents he small spheres (yellow) represent S atoms. Top view of a single layer 2H-TaS$_2$ superlattice. **b** Schematic illustration of the unit-cell structure. The bulk 2H structure contains two trilayers per unit cell.

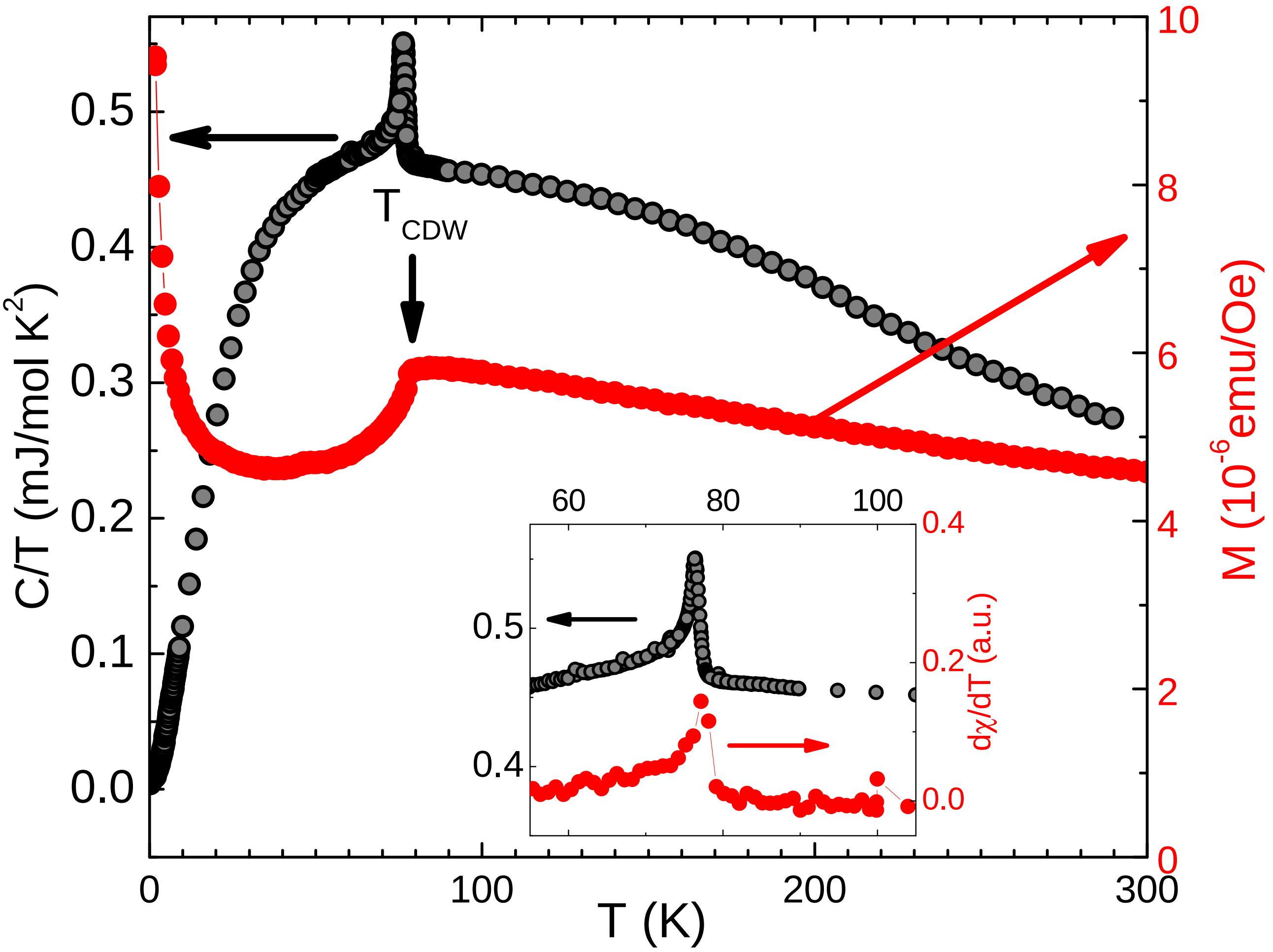

Extended Data Figure 2: Temperature dependence of the magnetic susceptibility and specific heat 2H-TaS$_2$. The right side presents the T-dependence of the magnetization measured under 0.5 T parallel to the crystallographic basal plane. The left side shows the specific heat data measured in zero field conditions. The inset: enlarged C_p/T vs. T plot and the derivative of the magnetization near the CDW transition.

the S atom in the layer beneath, but the lateral registry is different: in 2H the S atoms are fully eclipsed. The inset of Extended Data Figure 1**a** presents top view of a single layer 2H-TaS$_2$ superlattice, while the inset **b** illustrates the unit-cell structure of 2H-TaS$_2$. The tantalum atoms are surrounded by sulfur atoms following a hexagonal coordination.

In order to examine the composition of 2H-TaS$_2$ single crystals, an electron probe micro analysis (EPMA) system was used. Bulk analysis of the EPMA shows that TaS$_2$ has a composition of 1 : 2.0066, which is in excellent agreement with the composition of the investigated system. To determine the crystalline structure of 2H-TaS$_2$, we have done X-ray diffraction measurements on our single crystal from the same batch as those measured with transport, magnetization and specific heat using a Bruker D8 VENTURE Single Crystal X-ray Diffractometer, see the Extended Data Figure 1. We find a clear evidence for the purity in our system and the XRD pattern shows sharp distinct peaks.

The temperature dependence of specific heat and magnetization are illustrated in Extended Data Figure 2. A clear maximum of both data at 76 K, typically found in 2H-TaS$_2$ which is weakly first-order, is an indication of the CDW transition. The CDW transition temperature is in good agreement with transport and earlier reports. A jump in the specific heat at 76 K of 10.14 J/mol K is found (full width at half maximum $\approx$ 1.2, and 1 K). This transition is sharper than previously on polycrystalline samples [1] and indicates the high quality of our single crystals. On the other hand, below the CDW transition the magnetic susceptibility decreases sharply and continuously. This decrease in the susceptibility can be attributed to the decrease in the density of states on the hole band due to the opening of the CDW gap and additionally there is no long-range magnetic order was ever detected in this material [2].

ELECTRONIC SPECIFIC HEAT

We discuss various gap values obtained in the present simple system for analyzing the T-dependence of our zero-field specific heat data down to 70 mK. The normalized $C_{\rm el}/\gamma_n T$ as a function of the reduced temperature $T/T_{\rm c}$ is presented in Extended Data Figure 3 together with the fits to various models. It is obvious that the superconducting transition at $T_{\rm c}$ is well pronounced, with a sharp jump in $C_{\rm el}$ at T_c. We have attempted best fits to the data using three different models: the single-band weak-coupling BCS theory with the s-wave gap $\Delta_{(0)}/k_BT_c = 1.76$; a d-wave calculation[3] using $\Delta = \Delta_{(0)}\cos(2\phi)$; and a two-gap s-wave. The almost linear T-dependence of $C_{\rm el}/\gamma_n T$ indicates that the experimental data cannot be described by the standard BCS-model. Below T_c we observed a systematic deviation of both single-gap and d-wave fits from the data showing a higher jump at T_c than the s-wave model. Thus, we focus our discussions on the possibility of two superconducting energy gaps using the generalized α-model explaining the specific heat behavior in a multi-band superconductor [4]. Although the two-gap model contains two distinct gaps, the specific heat value is calculated as the sum of contributions, each one following the BCS-type T-dependence, $\Delta(0) = \gamma_1\Delta_s(0) + \gamma_2\Delta_l(0)$ [4]. The thermodynamic properties are obtained as the sum of the contributions from the individual bands, i.e., $\alpha_s = \Delta_s/k_BT_c$ and $\alpha_l = \Delta_l/k_BT_c$. The estimated $\Delta_s(0)/k_BT_c$ for the small gap is 1.2±0.1, while the large gap $\Delta_l(0)/k_BT_c$ is found to be 2.05±0.1 and the calculated data are illustrated by the solid line. The error bars represent the width of the corresponding range of gap amplitudes obtained in the fits for both values of $\Delta_s(0)/k_BT_c$ and $\Delta_l(0)/k_BT_c$. The relative weight of each contribution illustrates that $\gamma_2/\gamma_n = 0.68$ is larger than $\gamma_1/\gamma_n = 0.32$. This indicates that the major gap develops around the FS sheet that exhibits the largest density of states. The ratio of the two gaps $\Delta_s(0)/\Delta_l(0) \approx 0.58$ is comparable to the $NbSe_2$ case [5]. It has been theoretically demonstrated that it is a multiband superconductor if the ratio of two isotropic s-wave gaps $\Delta_1(0)/\Delta_2(0) > 0.5$ [6] as in our case, further confirming the multi-band nature in $2H$-TaS_2.

BAND STRUCTURE AND ELECTRONIC DOS

The obtained band structure for $2H$-TaS_2 is displayed in Extended Data Figure 4**a**. The Fermi surface is shown in Extended Data Figure 4**b** and the projected density of states for Ta-d orbitals and S-p orbitals on a per-atom basis in Extended Data Figure 4**c**. For the band structure, it is obvious that two bands cross the Fermi level in the energy range from -1 eV to 2 eV and contribute to the conductivity. These two bands give rise to Fermi surface as depicted in **b**, which further suggests the multi-gap nature in our system. Correspondingly in the projected density of states shown in **c**, we find that the states near the Fermi level are mostly derived from d_{xy}, d_{z^2} and $d_{x^2-y^2}$ orbitals of tantalum and p orbitals of sulfur have also a contribution to the states. The metal d and p contributions are estimated to be 0.531 states/eV and 0.074 states/eV, respectively. Interestingly, the width and the peaks of the projected density of states are almost coincidentally same, which is due to the formation of the strong Ta-S covalent bonds. This strong covalent effect as well as the superconductivity instability nature of the Fermi surface of a transition metal suggests that the electrons involved in pairing in bands are primarily derived from d states, hybridized with p states.

In summary, based on both experimental and theoretical studies, we believe that the multi-gap scenario is more favorable than the anisotropic s-wave model in describing the gap structure of $2H$-TaS_2.

[1] Craven, R. A. & Meyer, S. F. Specific heat and resistivity near the charge-density-wave phase transitions in $2H$-$TaSe_2$ and $2H$-TaS_2. *Phys. Rev. B* **16**, 4583 (1977).

[2] Wilson, J. A. DiSalvo, F. J. & Mahajan, S. Charge-density waves and superlattices in the metallic layered transition metal dichalcogenides. *Adv. Phys.***24**, 117 (1975).

[3] Volovik, G. E. Superconductivity with lines of the gap nodes: Density of states in the vortex. *JETP Lett.* **58**, 469 (1993).

[4] Bouquet, F. *et al.* Phenomenological two-gap model for the specific heat of MgB_2. *Europhys. Lett.* **56**, 856-862 (2001).

[5] Huang, C. L. *et al.* Experimental evidence for a two-gap structure of superconducting $NbSe_2$: A specific-heat study in external magnetic fields. *Phys. Rev. B* **76**, 212504 (2007).

[6] Bang, Y. Volovik effect in the s-wave state for the iron-based superconductors. *Phys. Rev. Lett.* **104**, 217001 (2010).

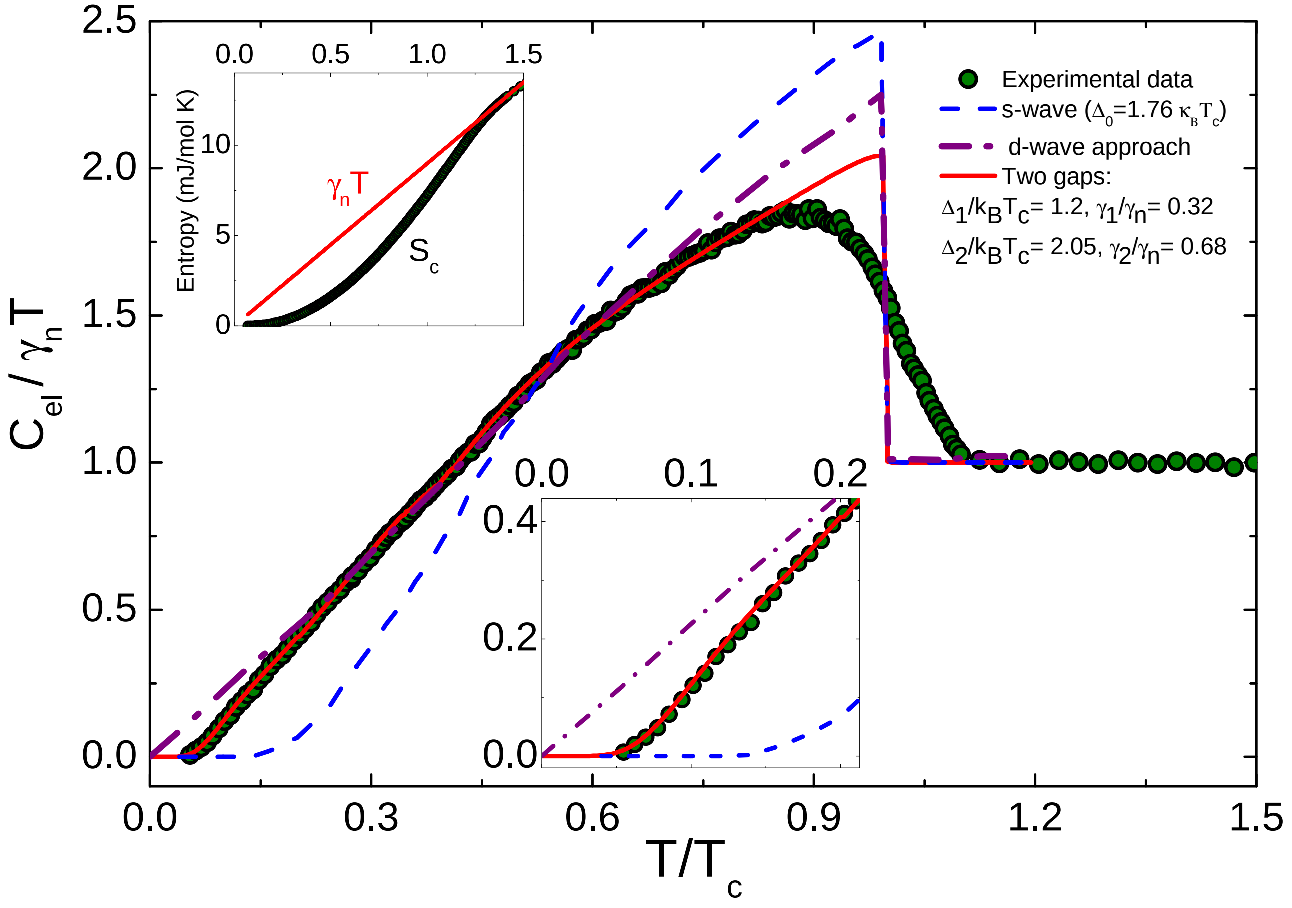

Extended Data Figure 3: Electronic contribution to the specific heat of 2H-TaS$_2$. The normalized C_{el} after subtracting the phonon contribution as a function of reduced temperature, T/T_c. The dashed line represents the theoretical curves based on single-band weak-coupling BCS theory, while the dotted line illustrates the d-wave approximation. The solid red line indicates the curve of the two s-wave gap model. The lower inset shows the low-T data on a larger scale. The upper inset illustrates the entropy in the normal and superconducting state as a function of temperature.

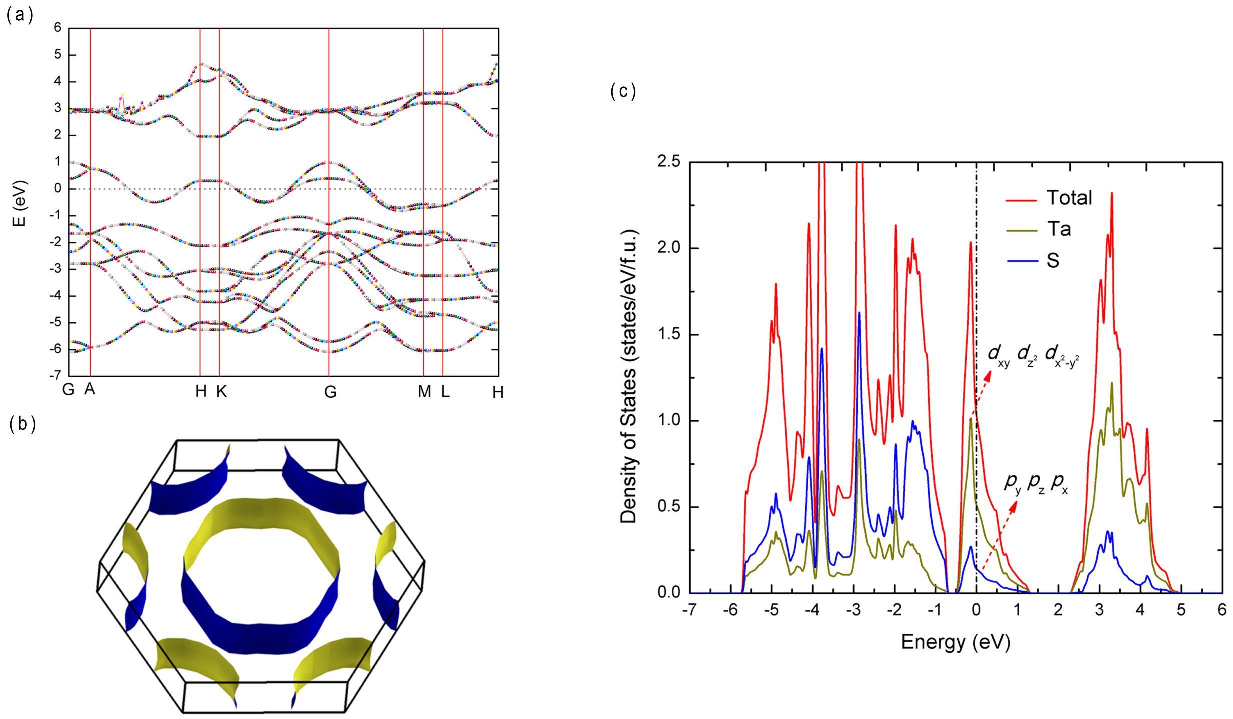

Extended Data Figure 4: Calculated band structure and electronic DOS of 2H-TaS$_2$. **a** The band structures for 2H-TaS$_2$ **b** The three-diemensional (3D) Fermi surface in the first Brillouin zone. **c** The density of states projected for both tantalum and sulfur atoms in the unit-cell.
